# Photobiomodulation increases brain metabolic activity through a combination of 810 and 660 wavelengths: a comparative study in male and female rats

**DOI:** 10.1007/s10103-023-03966-0

**Published:** 2024-01-12

**Authors:** Candela Zorzo, Lucía Rodríguez-Fernández, Juan A. Martínez, Jorge L. Arias

**Affiliations:** 1 Neuroscience Laboratory, Department of Psychology, University of Oviedo, Oviedo, Spain; 2 INEUROPA, Instituto de Neurociencias del Principado de Asturias, Oviedo, Spain; 3 ISPA, Instituto de Investigación Sanitaria del Principado de Asturias, Oviedo, Spain; 4 Electronic Technology Area, University of Oviedo, Gijón, Spain

**Keywords:** Photobiomodulation, Cytochrome c-oxidase, Brain metabolism, Red light; infrared light; wavelength

## Abstract

Photobiomodulation (PBM), an emerging and non-invasive intervention, has been shown to benefit the nervous system by modifying the mitochondrial cytochrome c-oxidase (CCO) enzyme, which has red (620–680 nm) or infrared (760–825 nm) spectral absorption peaks. The effect of a single 810-nm wavelength with a combination of 810 nm and 660 nm lights in the brain metabolic activity of male and female rats was compared. PBM, with a wavelength of 810 nm and a combination of 810 nm and 660 nm, was applied for 5 days on the prefrontal cortex. Then, brain metabolic activity in the prefrontal area, hippocampus, retrosplenial, and parietal cortex was explored. Sex differences were found in cortical and subcortical regions, indicating higher male brain oxidative metabolism, regardless of treatment. CCO activity in the cingulate and prelimbic area, dentate gyrus, retrosplenial and parietal cortex was enhanced in both treatments (810 + 660 nm and 810 nm). Moreover, using the combination of waves, CCO increased in the infralimbic area, and in CA1 and CA3 of the hippocampus. Thus, employment of a single NIR treatment or a combination of red to NIR treatment led to slight differences in CCO activity across the limbic system, suggesting that a combination of lights of the spectrum may be relevant.

## Introduction

Photobiomodulation (PBM) is a promising tool for stimulating, healing, regenerating, and protecting damaged tissues through the use of the red to near-infrared (NIR) spectrum (600–1200 nm) [1, 2]. Its medical implication was discovered in 1967, when Endre Mester observed that PBM promoted hair regrowth and wound healing in rats [3]. Since its discovery, PBM has been recognised by many organisations, researchers, and clinicians in the biomedical field, as this technique can repair damaged tissues, restore brain blood flow, minimise inflammation, stimulate neurogenesis, synaptogenesis, and nerve growth factors [4, 5], among other effects. Consequently, it has been employed in a wide range of alterations that affect the nervous system; for example, for the treatment of traumatic events such as stroke, traumatic brain injury, or ischemia, in degenerative diseases and psychological/psychiatric alterations, and even to prevent cognitive decline in healthy ageing [1, 6–10].

PBM action mechanism affects an enzyme of the mitochondrial respiratory chain, cytochrome c oxidase (CCO). The CCO enzyme is located in the IV complex of the electron transport chain, in the inner mitochondrial membrane. It catalyses the final reduction of molecular oxygen (O_2_) into two water molecules (H_2_O), using the electrons generated in glucose metabolism, and pumps protons out of the matrix. This pumping, in turn, generates energy that produces ATP synthesis [11]. Brain functioning is critically dependent on oxygen consumption by CCO for ATP production, which can be examined by CCO histochemistry.

Interestingly, the CCO enzyme is the major intracellular photoceptor that can absorb specific wavelengths, leading to molecular and cellular modifications [1, 12]. The CCO enzyme contains a heme and copper centres with red (620–680 nm) or infrared (760–825 nm) spectral absorption peaks [13]. However, CCO’s absorption of light is not limited to these wavelengths, as it has been shown that longer NIR wavelengths, such as 1064, can trigger a hemodynamic response, leading to greater brain oxygenation [13, 14]. When CCO absorbs photons derived from a PBM device, ATP synthesis is enhanced due to an increase in the mitochondrial reactive oxygen species (ROS), which activates signalling pathways associated with protective, antioxidant and antiapoptotic effects in the cells. The dissociation of nitric oxide (NO) after light stimulation increases mitochondrial membrane potential, oxygen consumption, and glucose metabolism [4, 13]. Production of ROS and release of Ca2 + may follow this process, leading to the activation of transcription factors and signalling mediators with long-lasting effects on cells [15]. The signalling pathways can also modulate the long-term expression of various proteins and genes. The stimulation of CCO also induces the replication of mitochondrial DNA, thereby activating early genes [4, 13]. Therefore, PBM emerges as a novel approach to modulating bioenergetics in the brain [12, 16].

Many PBM parameters such as wavelength, wavelength type (continuous or pulsated), frequency, intensity, irradiance, brain target area, and days of application can have different effects [6, 17], and need to be considered. In this study, we focused on the wavelength, where 810 nm is thought to be more effective, as it promotes greater light absorption [18]. Specifically, it has been shown that NIR light penetrates tissue more deeply (> 30–40 mm), whereas red light penetrates up to < 10 mm [19]. Therefore, we aim to determine the effect of a single NIR (810 nm) PBM treatment and a combination of NIR (810 nm) and red (660 nm) PBM application on brain oxidative metabolism through COO histochemistry in healthy adult male and female rats.

## Material and methods

### Animals

A total of 24 male (275–315 gr at the beginning of the experiment) and 24 female (225–300 gr) Wistar rats were used. All the animals had ad libitum access to food and tap water and were maintained at constant room temperature (20-22ºC), with a relative humidity of 65–70% and an artificial light–dark cycle of 12 h (08:00–20:00/20:00–08:00 h). The animals were caged in groups of four rats in transparent polycarbonate cages.

The procedures and manipulation of the animals followed the European Communities Council Directive (2010/63/UE) and the Spanish legislation on the care and use of animals for experimentation (RD 53/2013). The local committee for animal studies of Oviedo University approved the study (PROAE 23/2021).

We applied PBM for 5 consecutive days with a wavelength of 810 nm (810 male and female groups) and, for 5 consecutive days, a combination of 810 nm and 660 nm, emitting 810 nm on 3 days and 660 nm on 2 days (810 + 660 male and female groups). Male and female rats were randomly split into 6 groups: control male group (CM, *n* = 8), 810 nm PBM male group (810 M, *n* = 8), 810 nm + 660 nm PBM male group (810 + 660 M, *n* = 8), control female group (CF, *n* = 8), 810 nm PBM female group (810 F, *n* = 8), and 810 nm + 660 nm PBM female group (810 + 660 F, *n* = 8).

### Photobiomodulation therapy

#### Apparatus

The lasers were adjusted to provide an optical power of 40 mW. The lasers are cased inside a cylinder 45 mm high and 18 mm in diameter. The case also holds the driver for the laser diodes and a 7.50-mm lens. As the devices used produce a dot of 2.43 mm in diameter, the irradiance externally applied externally is 862 mW/cm^2^. Previous experiments have demonstrated than only 0.8% of the external irradiance actually reaches the test animal’s brain. Therefore, only 6.9 mW/cm2 were applied on the area of interest. The lasers were operated in cycles of 40 s ON followed by 10 s OFF to prevent excessive device heating. These cycles were applied at a rate of 14 cycles/day for 5 days, resulting in a total energy delivered of approximately 20 J/cm2. For the 810 nm male and female groups, the energy delivery was 4 J/cm2 each day, leading to 20 J/cm2 for the entire treatment. For the 810 + 660 nm male and female groups, the energy was applied using the 810-nm wave for 3 days (resulting in an energy delivery of 12 J/cm^2^) and the 660-nm wave for 2 days (providing the remaining 8 J/cm^2^). The pattern of the waveforms to be applied is generated by an external microcontroller-based circuit. Table 1 summarises the parameters employed.

**Table 1:** PBM parameters selected for each wavelength

	810 nm	660 nm
Wave type	50-s cycles (40 s ON and 10 s OFF)	
Duration	30 min (3 blocks × 10 min each; ITI 30 min)	20 min (2 blocks × 10 min each; ITI 20 min)
Irradiance	69 W/m^2^	
Output optical power	40 mW	
Fluency	4 J/cm^2^ each day	
Target area	Prefrontal cortex	Prefrontal cortex

The apparatus was previously calibrated using a PM160 optical power meter from Thorlabs. The laser driver was adjusted until the power meter showed that it provided 40 mW. The power meter used was calibrated by the manufacturer (calibration certificate number 15239113314). The 810-nm device has a laser diode with a rated output power of 500 mW at 50 °C maximum (S810500MG, *Roithner Lasertechnik*), and the 660 nm has a laser diode with a rated output power of 100 mW at 85 °C maximum (LNCQ28PS01WW, *Panasonic*).

#### Treatment protocol

**Fig. 1:**
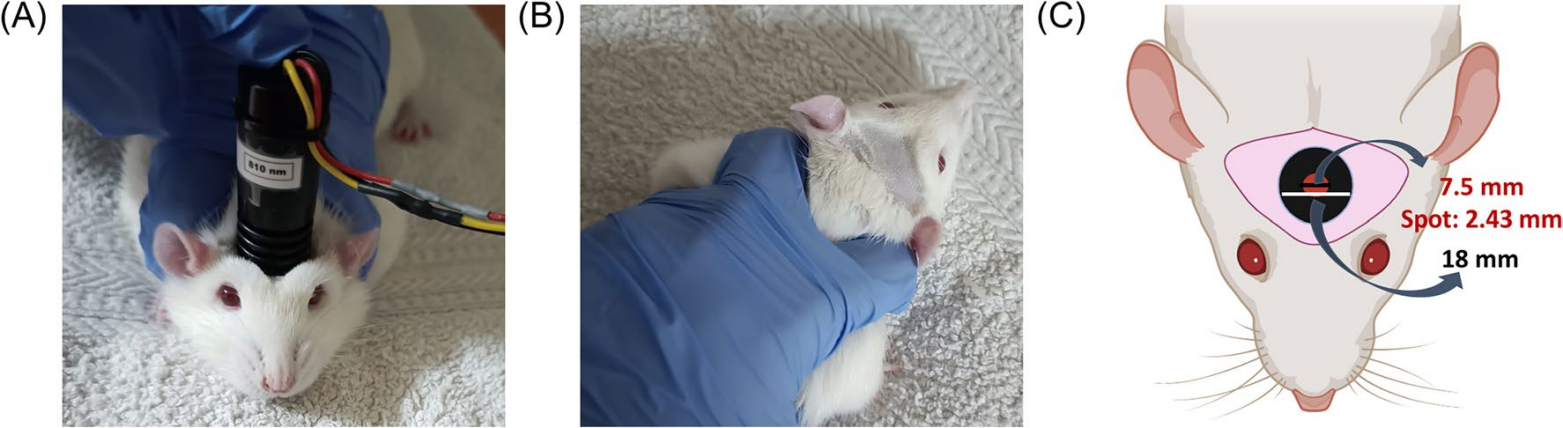
PBM application. (**A**) Immobilisation procedure and PBM application (**B**) Subject’s shaved head. (**C**) Diagram of the diameters of the device and irradiation point (spot). The device is applied directly on the scalp between the ears and eyes

Before PBM treatment, the rats were habituated to the researcher and the immobilisation required for the treatment for one week. They were shaved across prefrontal areas to maximise light penetration. The target area for PBM was the prefrontal cortex, and the PBM device was transcranially placed at this location (Fig. 1).

**Fig. 2:**
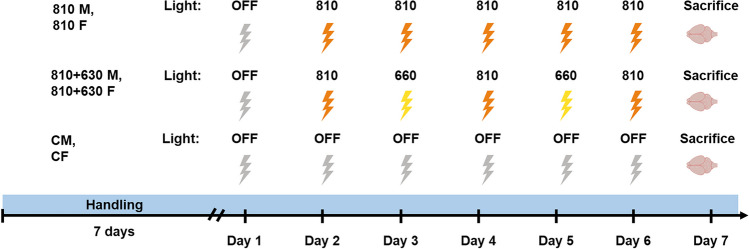
Experimental design of PBM application

The therapy lasted for 1 habituation day and 5 consecutive PBM active days. On these days, subjects were immobilised by the researcher on a soft surface while the PMB device was placed on the shaved region. For the 810-nm male and female groups, the treatment began with this immobilised procedure, but the light device was in ON mode. A laser with a continuous wave at 810-nm wavelength was used for these groups for 5 consecutive days. For the 810 + 660 nm male and female groups, immobilisation was carried out identically, but rats received PBM with a laser with a continuous wave at 810 nm for 3 days (days 1, 3, and 5), and at 660 nm for 2 days (days 2 and 5). Control groups were immobilised with the same procedure for 5 consecutive days, but the device was in OFF mode, as in habituation (Fig. 2). PBM was administrated in three blocks of 10 min, a total duration of 30 min per day. Intertrial interval (ITI) was 30 min. During habituation, the device was set to OFF mode.

### Tissue processing

The day after finishing the light procedure, the animals were decapitated, and the brains were removed intact, frozen rapidly in isopentane, and stored at -40 ºC. Coronal Sects. (30 µm) of the brain were cut at -20 ºC in a cryostat and mounted on non-gelatinised slides for cytochrome c oxidase (CCO) histochemistry. The regions of interest were anatomically defined according to Paxinos and Watson’s atlas [20].

### CCO histochemistry and quantification

Section slides were processed with quantitative CCO histochemistry, described by Gonzalez-Lima and Cada [21].  To quantify enzymatic activity and control staining variability across the baths, sets of tissue homogenate standards from adult Wistar rat brains were cut at different thicknesses. The sections and standards were incubated for 5 min in 0.1 phosphate buffer with 10% sucrose and 0.5 glutaraldehyde, pH 7.6. Afterwards, they were bathed with a 0.1 M phosphate buffer with sucrose performed for 5 min each. Subsequently, 0.05MTris buffer, pH 7.6, with 275 mg/l cobalt chloride, sucrose, and 0.5 dimethylsulfoxide were applied for 10 min. Then, sections and standards were incubated in a solution with 0.0075% cytochrome-c, 0.002% catalase, 5% sucrose, 0.25% dimethylsulfoxide, and 0.05% diaminobenzidine tetrahydrochloride, in 0.1 M phosphate buffer at 37 °C for 1 h. The reaction was interrupted by fixing the tissue in buffered 4% formalin for 30 min at room temperature. Finally, the slides were dehydrated, cleared with xylene, and coverslipped.

The CCO histochemical intensity was quantified by densitometric analysis, using a computer-assisted image analysis workstation (MCID, Interfocus Imaging Ltd., Linton, England) consisting of a high-precision illuminator, a digital camera, and a computer with the specific image analysis software MDCID Core 7.0. The mean optical density (OD) of each region was measured using 3 consecutive sections for each subject. In each section, four non-overlapping readings were taken, using a square-shaped dissector adjusted for each region size. OD values were converted to CCO activity units, determined by the enzymatic activity of the standards measured spectrophotometrically. For CCO histochemistry, the regions studied were included in the bregma coordinates + 3.24 mm for the cingulate cortex (CG), prelimbic cortex (PL), and infralimbic cortex (IL), and in -3.24 mm for the CA1, CA3, and the dentate gyrus (DG) subfields of the dorsal hippocampus, granular retrosplenial cortex (RSG), disgranular retrosplenial cortex (RSD), and parietal cortex (PAR).

### Statistical analysis

All data were analysed with the Sigma-Stat 14 program (Systat, Richmond, USA). The results were considered statistically significant if* P* < 0.05. A two-way analysis of variance (ANOVA) was performed to explore differences between the PBM treatment (control, 810 + 660, 810) and sex (male, female) in each brain region. To assess multiple comparisons, the Holm-Sidak method was employed. When an interaction effect was found, post-hoc multiple comparisons considering the interaction of two factors were performed. When no interaction effect was found, but there were differences in the main effects, post-hoc analysis considering the significant factors was performed. Power analysis (1 – β) was calculated with alpha 0.05 and was described when significant differences were found. Graphic representation of the results was performed with the SigmaPlot 14 software program. Data are expressed as mean ± standard deviation (SD).

## Results

**Fig.3:**
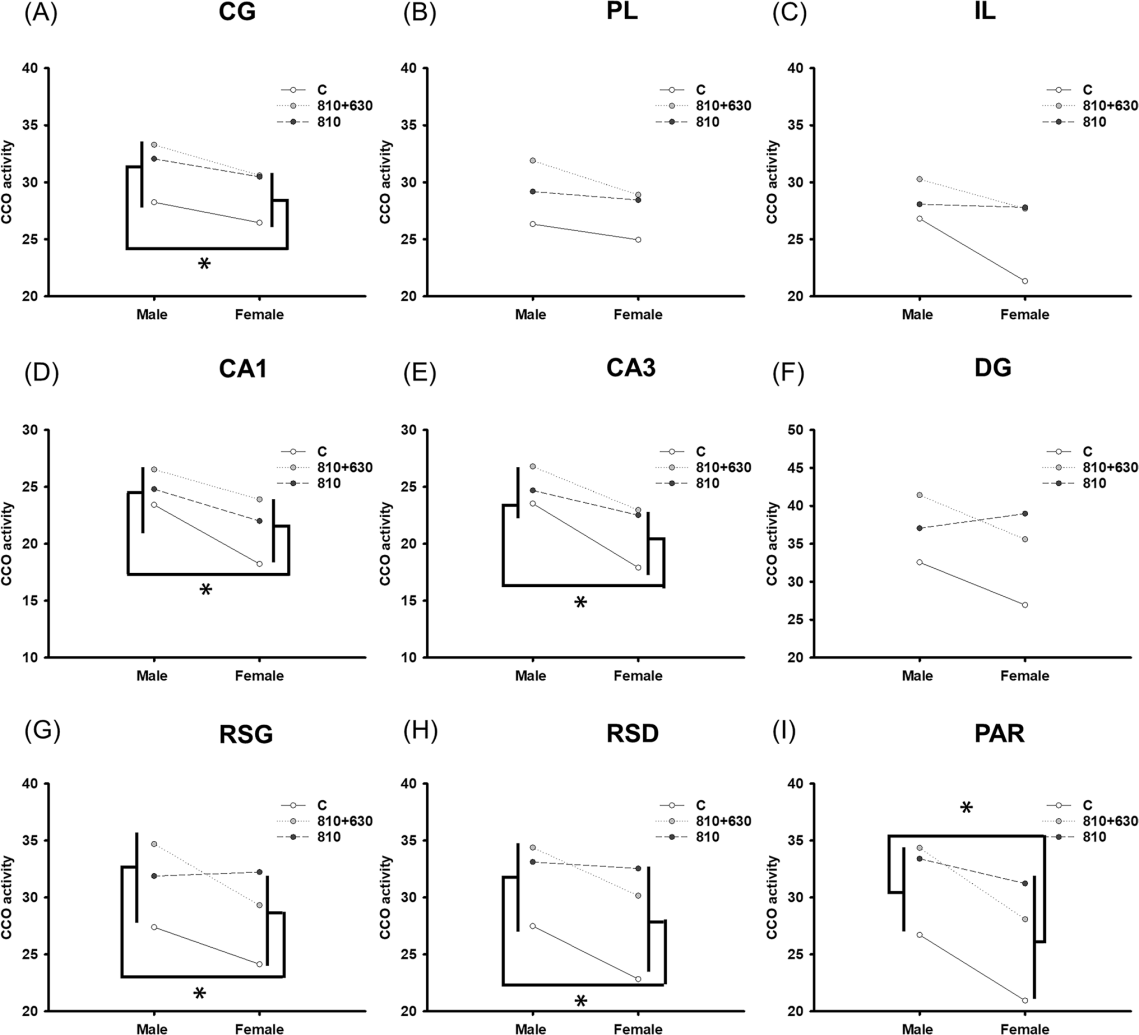
CCO activity of male and female rats in prefrontal cortex (**A**, **B**, **C**), hippocampus (**D**, **E**, **F**), retrosplenial (**G**, **H**), and parietal cortex (**I**). * Represents sex differences. There was no Treatment x Sex interaction effect

The analysis of metabolic brain activity revealed no effect of Treatment x Sex interaction in any brain area: CG (*F*_(2, 42) =_ 0.114, *P* = 0.892), PL (*F*_(2, 38) =_ 0.546, *P* = 0.584), IL (*F*_(2, 38) =_ 0.726, *P* = 0.490), CA1 (*F*_(2, 40) =_ 0.372, *P* = 0.692), CA3 (*F*_(2, 41) =_ 0.311, *P* = 0.734), DG (*F*_(2, 41) =_ 1.453, *P* = 0.246), RSG (*F*_(2, 40) =_ 2.692, *P* = 0.080), RSD (*F*_(2, 41) =_ 1.104, *P* = 0.341), PAR (*F*_(2, 41) =_ 1.552, *P* = 0.224). Thus, considering the main factors, sex differences were revealed regardless of treatment in CG (*F*_(2, 42) =_ 4.939; *P* = 0.032, β = 0.481), CA1 (*F*_(1, 40) =_ 14.223; *P* < 0.001, β = 0.959), CA3 (*F*_(1, 41) =_ 12.220; *P* = 0.001, β = 0.922), RSG (*F*_(1, 40) =_ 7.555; *P* = 0.009, β = 0.711), RSD (*F*_(1, 41) =_ 6.595; *P* = 0.014, β = 0.637), and PAR (*F*_(1, 42) =_ 14.123; *P* < 0.001, β = 0.958). Multiple comparisons revealed a higher brain metabolic activity in male than in female groups (CG: *t* = 2.222, *P* = 0.032; CA1: *t* = 3.771, *P* < 0.001; CA3: *t* = 3.496, *P* = 0.001; RSG: *t* = 2.800, *P* = 0.009; RSG: *t* = 2.568, *P* = 0.014; PAR: *t* = 3.758, *P* < 0.001) (Fig. 3).

**Fig.4:**
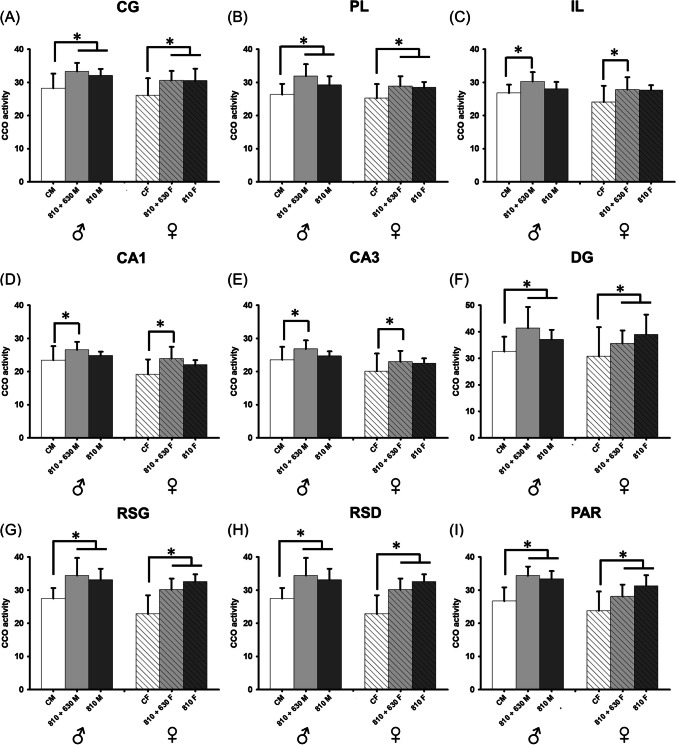
CCO activity of male and female rats in prefrontal cortex (**A**, **B**, **C**), hippocampus (**D**, **E**, **F**), retrosplenial (**G**, **H**), and parietal cortex (**I**). * Represents treatment differences. Data are expressed as mean ± SD

However, a treatment effect was found in CG (*F*_(2, 42) =_ 9.668, *P* < 0.001, β = 0.968), PL (*F*_(2, 38) =_ 8.329, *P* = 0.001, β = 0.931), IL (*F*_(2, 38) =_ 4.984, *P* = 0.012, β = 0.679), CA1 (*F*_(2, 40) =_ 6.881, *P* = 0.003, β = 0.860), CA3 (*F*_(2, 41) =_ 3.864, *P* = 0.029, β = 0.523), DG (*F*_(2, 41) =_ 5.796, *P* = 0.006, β = 0.772), RSG (*F*_(2, 40) =_ 17.617, *P* < 0.001, β = 1.000), RSD (*F*_(2, 41) =_ 18.793, *P* < 0.001, β = 1.000), and PAR (*F*_(2, 41) =_ 19.3268, *P* < 0.001, β = 1.000). Post-hoc analysis for factor treatment revealed differences in CG, PL, DG, RSG, RSD, and PAR between 810 + 660 (CG: *t* = 4.068, *P* < 0.001; PL:* t* = 4.001, *P* < 0.001; DG:* t* = 3.019, *P* = 0.013; RSG: *t* = 5.216, *P* < 0.001; RSD: *t* = 5.543, *P* < 0.001; PAR: *t* = 5.795, *P* < 0.001) and 810 groups (CG: *t* = 3.486, *P* = 0.002; PL:* t* = 2.669, *P* = 0.022; DG: *t* = 2.805, *P* = 0.013; RSG: *t* = 5.001, *P* < 0.001; RSD: *t* = 5.005, *P* < 0.001; and PAR: *t* = 4.808, *P* < 0.001), compared to controls. Differences were also found between 810 + 660 and controls in IL, CA1 and CA3 (IL: *t* = 3.059, *P* = 0.012; CA1: *t* = 3.696, *P* = 0.002; CA3: *t* = 2.761, *P* = 0.026) (Fig. 4).

## Discussion

Despite the promising studies on the benefits of PBM on health and disease conditions, its action mechanism is still not completely understood. In the present study, we explored the effect of differential PBM wavelength applications on brain metabolic activity in healthy male and female rats.

PBM action mechanisms are based on light penetration, which depends on the absorption and dispersion of the molecules present in the tissue. Penetration decreases as the wavelength increases, and with 810 nm being considered optimal [22], a wavelength included in both PBM treatments. Brain tissue chromophores can absorb photonic energy, leading to changes in cell metabolism and brain physiology [13, 23]. As noted above, the CCO enzyme is composed of 13 protein subunits containing two heme and copper centres with red (620–680 nm) and NIR (760–825 nm) absorption peaks, such that PBM oxidises this enzyme, accelerating and increasing oxygen consumption and ATP synthesis. When light reaches CCO and is absorbed by the enzyme, electrons are excited. It has been suggested that PBM in the red and NIR wavelengths induce NO photodissociation [15]. Thus, PBM can dissociate NO from CCO, leading to an enhancement of CCO activity. It has been reported that NO binds to the heme iron and copper centres of CCO and consequently reverses the inhibition of the electron transport chain [24]. This leads to an enhancement of mitochondrial membrane potential and an increase in oxygen consumption, ATP synthesis, and glucose metabolism rate [1, 13, 25]. These events are followed by ROS induction and a release of Ca^2+^, resulting in the activation of transcription factors and signalling pathways associated with cytoprotective, antioxidant and antiapoptotic effects [1, 13, 25].

Previous studies have outlined the need to explore PBM differences regarding sex [26–29] because male and female subjects may respond differentially to PBM treatments because of light penetration through the skull [26]. It is essential to analyse the effect of PBM including sex as an independent variable, to avoid sex and gender bias in neuroscience research [26, 30]. To address this issue, analyses were performed considering treatment and sex as the main factors and also the interaction effect between them, aligned with previous studies [28]. No Sex x Treatment interaction effect was found in any brain region, but analyses revealed sex differences, showing that CCO activity in CG, CA1, CA3, RSG, RSD, and PAR is higher in males. This effect includes the controls and both PBM treatments, suggesting that increased metabolic activity across several brain limbic regions in males appears, regardless of treatment. In this line, the study of Mällo et al. (2009) revealed sexual dimorphism, with males displaying a higher CCO activity under control and stress conditions [31], similar to [32]. Also, CCO expression has been found to differ between sexes in several behavioural tasks [28, 33–35], emphasising the need to include females in experimental and preclinical studies to determine whether they respond differently.

Regarding treatments, we observed an enhancement of CCO activity using the 810 + 660 nm PBM treatment in prefrontal areas (CG, PL, IL), hippocampus (CA1, CA3, DG), retrosplenial cortex (RSG, RSD), and parietal cortex in the male and female groups, compared to controls. Moreover, higher brain metabolic activity was found with 810 nm in the prefrontal cortex (CG, PL), hippocampus (DG), retrosplenial (RSG, RSD), and parietal cortex. The results reflect that both treatments increased oxidative metabolism in several brain regions, but more brain regions are engaged in the combination of wavelengths, suggesting differential mechanisms. The combination of 660 and 810 nm may result in appropriate neuromodulation of brain oxidative metabolic activity, as CCO activity was up-regulated in a large number of brain areas. This may be attributable to a summative effect across the heme and copper centres of the CCO enzyme by the two wavelengths within the spectrum. When applying only the NIR spectrum, there was also an increase in brain metabolic activity in male and female rats in some areas functionally interconnected with the prefrontal cortex [36]. In this line, it has been shown that the effect observed in the CCO activity due to PBM treatment is not limited to the target area stimulated, but instead spreads over several brain regions interconnected with the target [12, 37, 38]. However, it is important to note that no statistical differences were found between treatments, although there was an increase in 6 brain areas after 810-nm PBM treatment, and in 9 brain areas after a combination of waves.

To our knowledge, there are no animal studies comparing the effect of a single wavelength or a combination of wavelengths that conform the range of red to the NIR spectrum on brain activity in healthy subjects. Under pathological conditions (model of anxiety and depression), one study compared the therapeutic effect of a NIR wave (810 nm) with the red wave (660 nm). They observed that both treatments restored glucose levels (higher under stress), but the NIR light was more effective than the red laser in reducing immobility time in stressed animals [39]. The red light reduced cortisol levels, suggesting that both lights contribute differently at behavioural and molecular levels. We underline that the present study was performed with healthy adult male and female rats whereas [39] applied PBM to rats suffering from chronic stress, which are different from a neurobiological and behavioural perspective. However, stress may mediate the PBM treatment due to immobilisation [40], suggesting that the stress associated with the PBM procedure may be modulated by the red wavelength [39], applied only in the 810 + 660 nm groups. In human studies, a PBM device was described with diodes with two different wavelengths, simultaneously applying different waves included within the range of red to the NIR spectrum [40–42], resulting in cognitive enhancements [40] and emotional improvements [42]. To note, studies addressing this issue select a device with a higher number of infrared diodes compared with red [40, 41]. Further research is needed to delve into wavelength interactions and their effect on the nervous system.

Most studies select a single wavelength for the entire treatment (for a review, see [6]). As mentioned, light absorption is possible due to CCO enzyme activity within the mitochondria, observing enhancement of ATP synthesis and of the activity of the complex IV in the prefrontal cortex with 808 nm [43], and enhancement of CCO activity in healthy rats with 670 nm [38]. The increased CCO activity marked by histochemistry that we observed in male and female brain areas with a single 810 nm application or combined with a red light (660 nm) is similar to previous studies regarding brain oxidative metabolism. Longer wavelengths can also impact CCO, as it was recently observed that a single 1064-nm treatment leads to an increase of CCO in several brain regions, lasting up to 4 weeks [12]. In addition, some human studies have found an increment in the rate of oxygen consumption, attributed to an increase in CCO expression [44], a more efficient prefrontal blood oxygen flux [45], or a brain-wide connectivity enhancement [46]. Moreover, it has been shown that PBM (810 nm) applied to healthy young adults can modify the brain activity of functionally active networks, showing the relevance not only of PBM parameters, but also of biological and individual factors [47].

Interestingly, we found increased CCO activity with the treatments in both target and distal brain regions, in line with previous studies reporting modifications of the brain metabolic activity in areas that are distal to the light source and suggesting brain network reorganisation [16, 37, 38]. We suggest that the changes in CCO activity outside the prefrontal cortex are a consequence of brain interconnections, considering that it is implausible for light penetrance to reach hippocampal areas or other cortical areas distal to the light device. This assumption is based on previous studies, which consider that light penetration depends on both wavelength and type of tissue, with the NIR spectrum achieving the highest penetration through the skull [48]. Some studies reflect that 810 nm can reach percentages of 39% in rats [49], and others report that PBM cannot exceed 10 mm of penetration, but it can affect deeper structures indirectly through pathways such as circulation [50].

Most studies are focused on exploring the benefits of PBM in human population. Indeed, it has been found that PBM can improve cognitive and emotional functioning in healthy subjects with 810 nm [51] and 850 nm [52, 53], although other studies found no differences from treatment with a single application of 810 nm [54]. Regarding brain activity, a 850-nm PBM treatment applied over the prefrontal cortex can change brainwaves, reducing the entire cortical delta waves, which may be linked to improved cognitive performance [52]. However, most of these studies focus on the neurocognitive benefits of PBM, both in healthy and clinical population, and few studies further explore the brain mechanism [6, 13]. Therefore, more studies in animals and humans are needed to discover the optimum PBM parameters, including wavelengths, pulse frequency, energy density or sessions, and to personalize PBM treatments [55].

One limitation of the study is the absence of a group that only received 660-nm PBM wavelength to contribute more knowledge about the red-light spectrum and its effect on CCO. Also, other parameters apart from wavelength may produce a biological effect. Thus, to better understand the PBM action mechanisms, it is necessary to delve into the type of light emitted, irradiance, frequency, fluency, wave type, and the mode of application (including target area, duration of sessions and treatments).

## Conclusion

The present findings support PBM as a potential treatment at the cellular level; both single-wave 810-nm treatment or a combination of 810- and 660-nm treatments can increase CCO activity in different brain areas that conform the limbic system of male and female rats. There was a marked effect on brain networks using alternate red (660 nm) and NIR (810 nm) waves across several cortical and subcortical areas, suggesting that a combination of lights of the spectrum may be interesting. The behavioural consequences and the molecular and cellular mechanisms should be explored in greater depth.
